# Precision prognostication in neuroblastomas via clinically validated E2F activity signatures

**DOI:** 10.3389/fimmu.2025.1612667

**Published:** 2025-06-17

**Authors:** Donghan Cai, Huihuang Xu, Shiwei He, Di Xu, Lizhi Li

**Affiliations:** ^1^ Shengli Clinical Medical College, Fujian Medical University, Fuzhou, Fujian, China; ^2^ Department of Pediatric Surgery, Provincial Clinical Medical College, Fuzhou University, Fuzhou, Fujian, China; ^3^ Population Medicine Research Institute, Public Health School of Fujian Medical University, Fuzhou, China

**Keywords:** neuroblastoma, E2F-related genes, prognosis, immune, drug sensitivity

## Abstract

**Background:**

Neuroblastoma (NB) is the most common extracranial solid tumor in children, with high-risk NB (HR-NB) exhibiting dismal survival rates due to aggressive biology and therapy resistance. E2F transcription factors (E2Fs) are pivotal regulators of cell cycle progression and immune modulation, yet their prognostic and therapeutic implications in NB remain underexplored.

**Methods:**

Using transcriptomic data from the GEO, TARGET, and E-MTAB-8248 cohorts, we identified E2F-associated molecular subtypes via consensus clustering. A prognostic signature was constructed via LASSO regression and validated for risk stratification. Immune infiltration, tumor mutation burden (TMB), and drug sensitivity were analyzed via the CIBERSORT, ESTIMATE, and GDSC databases.

**Results:**

Four E2F-related genes (MAD2L1, CDC25A, CKS2, and NME1) were used to construct a prognostic nomogram that stratified patients into high- and low-risk groups, with low-risk patients exhibiting superior overall survival (P < 0.05). Multivariate Cox regression confirmed that the model was an independent prognostic factor (P < 0.001). High-risk patients presented lower immune and stromal scores, reduced immune checkpoint expression, distinct immune cell infiltration patterns, and significant differences in mutation spectrum and drug sensitivity (P < 0.001).

**Conclusions:**

The E2F-related prognostic signature effectively stratifies NB patients by risk and provides potential biomarkers for prognosis and targeted therapy in HR-NB patients. The identified signature enhances patient stratification and provides insights into NB tumor biology, the immune landscape, and potential treatment strategies.

## Introduction

Neuroblastoma (NB) is a highly aggressive pediatric malignancy and the most common extracranial solid tumor in children, accounting for a significant proportion of pediatric cancer-related mortality (1). Among NB cases, high-risk neuroblastoma (HR-NB) is particularly challenging and is characterized by an unfavorable prognosis and high recurrence rates. The key prognostic factors include age ≥18 months, MYCN oncogene amplification, advanced International Neuroblastoma Staging System (INSS) stage (III/IV), and unfavorable histology (2). Despite continuous advancements in multimodal therapies, including surgery, chemotherapy, radiotherapy, and immunotherapy, the 5-year overall survival (OS) rate for HR-NB remains dismally low at approximately 30%-40% (1, 3). The urgent need for novel prognostic biomarkers and therapeutic strategies underscores the importance of refining risk stratification and guiding precision medicine.

E2F transcription factors (E2Fs) are essential regulators of cell cycle progression, apoptosis, and DNA replication and play a central role in oncogenesis and cancer progression. In NB, aberrant E2F activity has been linked to uncontrolled tumor cell proliferation, enhanced tumor aggressiveness, and adverse clinical outcomes. Dysregulated E2F expression disrupts the delicate balance between proliferation and apoptosis, driving tumor progression (4, 5). Furthermore, E2Fs are closely associated with the modulation of the tumor immune microenvironment (TIME), influencing immune infiltration patterns and facilitating immune evasion. HR-NB is often characterized by an immunosuppressive TIME, with low CD8+ T-cell infiltration and increased regulatory T-cell (Treg) activity, which fosters tumor progression and confers resistance to therapy (6–8).

Emerging research suggests that E2Fs play a pivotal role in immune regulation by interacting with immune checkpoint pathways and inflammatory signaling networks (9). Given the intricate interplay among E2Fs, immune modulation, and tumor proliferation, understanding their prognostic significance in NB remains a critical yet underexplored study area. Investigating the role of E2Fs in shaping the TIME could provide novel insights into potential therapeutic targets.

Recognizing the profound impact of E2Fs on both tumor proliferation and immune landscape dynamics, this study aimed to characterize E2F-associated prognostic signatures systematically. By constructing and validating an E2F-related prognostic model, we aimed to refine risk stratification and identify promising therapeutic avenues for HR-NB patients. Integrating these prognostic markers into existing clinical staging frameworks may increase the predictive accuracy and ultimately improve treatment outcomes for NB patients.

## Methods

### Data collection and mining of mRNA profiles

The mRNA expression matrix and related clinical information were obtained from the GSE49711 and GSE73511 cohorts sourced from the GPL platform. These datasets and the corresponding clinical information related to neuroblastoma (NB) were utilized as the training dataset. Data from the TARGET-NBL and E-MTAB-8248 cohorts were employed as the validation dataset, with corresponding clinical information sourced from the UCSC Xena platform (10). The TARGET-NBL cohort data played a crucial role in tumor mutation burden (TMB) analysis. The GSE49711 cohort, the TARGET-NBL cohort, and the E-MTAB-8248 cohort included 498, 143, and 223 NB patient samples, respectively. E2F-related genes were retrieved from the Molecular Signatures Database (MSigDB) and relevant literature.

### Unsupervised clustering of E2F-associated differentially expressed genes

The “ConsensusClusterPlus” R package, which is based on the k-means machine learning algorithm, was used to perform unsupervised consensus clustering. This method allows for dividing cases into multiple clusters based on the provided hallmarks or signatures. The set of E2F hallmark genes was acquired from the Molecular Signatures Database (MSigDB). Specifically, we used the consensus clustering algorithm with 1,000 iterations by sampling 80% of the data in each iteration. The optimal cluster number was confirmed by the item–consensus plot, the proportion of ambiguous clustering (PAC) algorithm, and the relative change in the area under the cumulative distribution function (CDF) curves. Two clusters were selected for assessing E2F status. Kaplan–Meier plots were generated for the two clusters to compare their overall survival (OS) rates.

### Determination and annotation of E2F-associated differentially expressed genes

By comparing the gene transcription profiles of patients from the training dataset with the R package “limma”, overall DEGs were identified (|fold change| > 1, p < 0.05). Pearson correlation was performed to select E2F-associated DEGs based on data from overall DEGs and E2F hallmark genes with the standards of Cor > 0.8 and P < 0.05. The potential functions of these E2F-associated DEGs were then ascertained through Gene Ontology (GO) annotation and Kyoto Encyclopedia of Genes and Genomes (KEGG) enrichment pathway analysis via the “clusterProfiler” package in R; FDR < 0.05 was considered statistically significant.

### Construction and validation of the E2F-associated prognostic signature

The intersection between E2F-associated DEGs and other relevant gene sets was determined, and the overlapping genes were selected for univariate Cox regression analysis. These genes were then processed with the least absolute shrinkage and selection operator (LASSO) to avoid overfitting and to delete tightly correlated genes. Tenfold cross-validation was employed to select the minimal penalty term (λ). An E2F-associated prognostic signature involving a set of E2F-associated DEGs was established. The formula of the risk score was constructed as follows:


$$\text{Risk score}=\sum_{\text{i}=1}^{\text{n}}{\text{Coef}}_{\text{i}}*{\text{x}}_{\text{i}}$$

where ${\text{Coef}}_{\text{i}}$ represents the coefficients and where ${\text{x}}_{\text{i}}$ represents the normalized count of each gene. Patients were stratified into high- and low-risk groups based on the median risk score. K–M analysis and survival-dependent receiver operating characteristic (ROC) curve analysis at 1, 2, and 5 years were performed in the training set and validated in the TARGET-NBL and E-MTAB-8248 cohorts.

### Independent prognostic value of the signature genes and their relationships with E2F clusters

The independent prognostic value of the signature genes was analyzed via univariate Cox regression analysis. The relationship between the risk score model and previously constructed E2F clusters was analyzed via the R package “pheatmap.” Kaplan–Meier plots of OS for these signature genes were also generated.

### Correlations between the E2F-associated gene signature and clinical parameters

Subgroup analysis of individual signature genes in the E2F-associated prognostic signature was conducted based on patients’clinical characteristics. Uni- and multivariate Cox regressions were used to verify the prognostic role of the E2F-associated gene signature and select clinical factors. A nomogram was established via the R package”rms”based on risk scores and clinical factors with prognostic value. The predictive effect of the nomogram was validated by assessing the discrimination and calibration plots.

### Gene set enrichment analysis of the prognostic risk score model

Gene set enrichment analysis (GSEA) was performed to determine the statistical significance of the identified molecular pathways and the heterogeneity between the high- and low-risk groups. The GSEA software was downloaded from the official website. The gene sets “h.all.v7.4.symbols.gmt” and “c5.go.v7.4.symbols.gmt” were selected as reference gene sets. A pathway with FDR Q < 0.25 and P < 0.05 was defined as statistically significant.

### Relationships of the prognostic gene signature with immune cell infiltration

Based on the RNA-seq expression matrix of NB, the CIBERSORT algorithm was applied to analyze differences in immune cell infiltration status between the high- and low-risk groups in terms of 22 immune cell subtypes. The ESTIMATE algorithm was used to measure the stromal level (stromal score), degree of immune cell infiltration (immune score), and tumor purity in the respective NB samples. Additionally, the expression status of common immune checkpoints was analyzed between the high- and low-risk groups via boxplots.

### Mutation analysis of the risk score model

Somatic mutation data were acquired from the TARGET-NBL cohort. The R package “maftools” was used to generate a waterfall plot to depict the mutation landscape in patients in the high- and low-risk groups.

### Statistical methods

Independent Student’s t tests were used to compare continuous data with a normal distribution, and the χ2 test was used for categorical data. The Kruskal–Wallis test was performed to determine statistically significant differences between multiple groups. The Mann–Whitney U test was used to compare differences between two independent groups when the dependent variable was either ordinal or continuous but not normally distributed. Kaplan–Meier analysis with a log-rank test was used to compare overall survival between different subgroups. All the statistical analyses were performed via the R programming language (version 4.4.0). A difference of P < 0.05 was considered statistically significant unless otherwise specified.

## Results

### Exploration of E2F-associated genes

**Figure 1:**
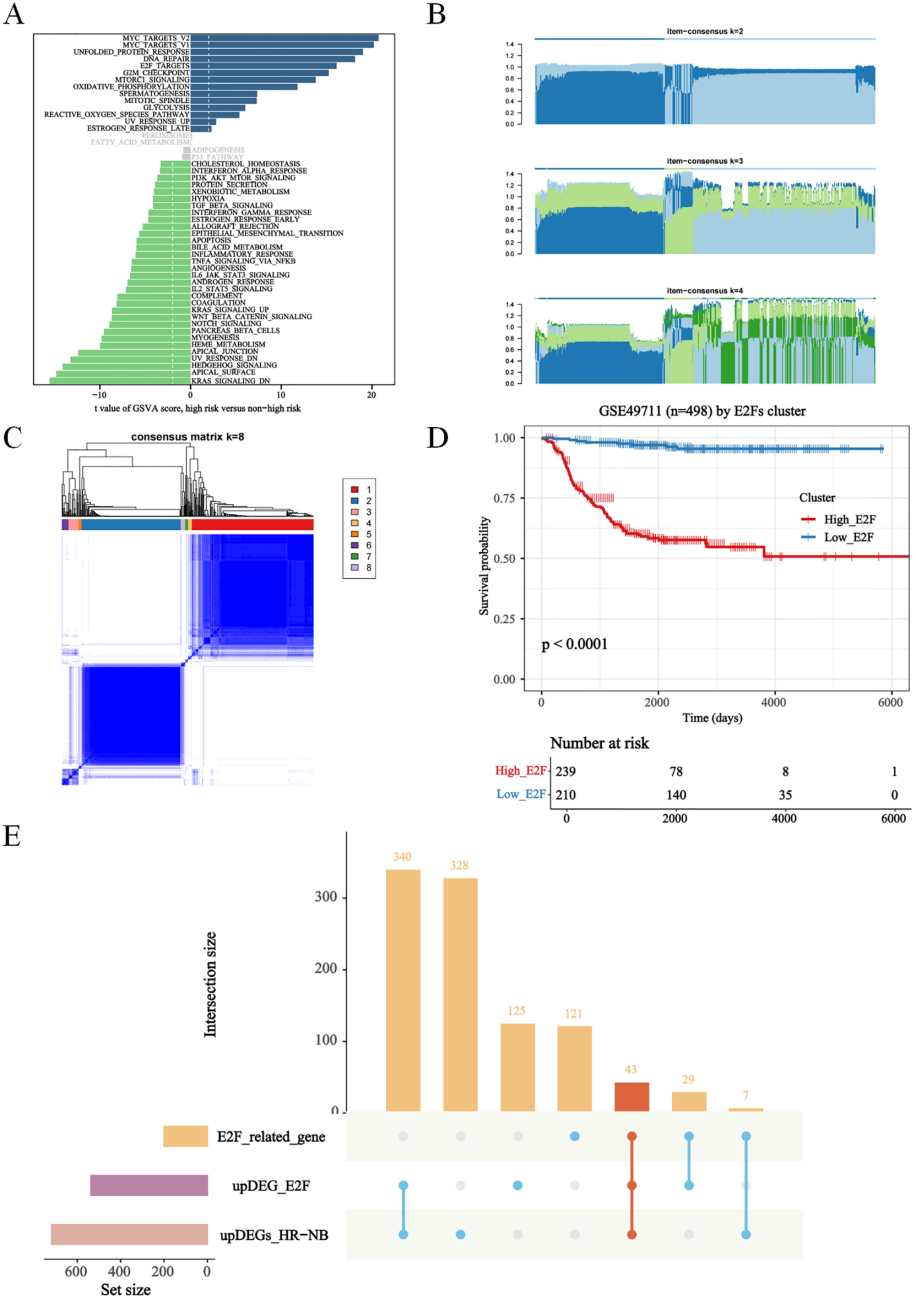
Exploration of E2Fs genes in distinct risk stage of NB. **(A)** The GSVA in distinct risk stage of NB (*P*<0.05); **(B)** The Item-Consensus Plot represented the chosen optimal cluster number (k = 2) for E2F genes. **(C)** The consensus matrix (k = 8) for E2Fs genes. **(D)** Survival curves of patients in E2Fs high and low. **(E)** Upset diagram showing overlapping DEGs among E2F_related gene, up-regulated DEGs in E2Fs-high group and up-regulated DEGs in HR-NB.

Significant differences in E2Fs were suggested based on the results of GSVA in NB patients with different risk stratifications (**Figure 1A**). Therefore, this study first performed an unsupervised cluster analysis on the GSE49711 dataset via the E2F gene set to identify different E2F patterns; we observed a range of consensus values from 0 to 1 (**Supplementary Figure S1A**) and a relative change in the area under the cumulative distribution function (CDF) curve when the number of clusters was varied from k to k+1 (**Supplementary Figure S1B**). k was varied in the range of 2–9, with the optimal value of k being 2 (**Supplementary Figure S1B**). Thus, patients were classified into two different risk stages for E2Fs (**Figures 1B, C**). A significant difference was detected between these two stages in terms of overall survival (OS) (**Figure 1D**), where patients with high E2F expression had a poorer prognosis than those with low E2F expression. This finding prompted us to continue exploring the relationship between the level of E2Fs and the prognosis of patients with NB, which was further analyzed by studying the expression of E2F-related genes.

A total of 43 genes were screened by taking the intersection of highly expressed genes in HR-WT, highly expressed genes in E2F clustering analysis, and E2F-related genes (**Figure 1E**). The Cox results for these genes are presented in **Table 1**.

**Table 1:** Unicox of E2Fs.

Gene	HR	Cl (5%-95%)	p value
NME l	2.58	2.15-3.l	' l .80E-24
CENPM	2.44	1.96-3.02	6.23E-16
BIRC5	2.16	1.83-2.55	8.55E-20
TOP2A	1.92	1.6-2.31	1.61E-12
PAICS	3.77	3.03-4.69	l .03E-32
KIF18B	1.98	1.65-2.38	3.82E-13
CHEKl	2.63	2.17-3.18	3.25E-23
CDKN3	2.71	2.26-3.25	6.04E-27
MKI67	2.19	1.81-2.66	6.88E-l 6
DLGAP5	2.7 1	2.22-3.31	9.98E-23
PLK4	3	2.4-3.74	2.37E-22
BUBl B	2.78	2.21-3.48	8.82E-19
CDC20	2.09	1.73-2.53	3.37E-14
E2F8	2.26	1.88-2.71	4.63E-18
MELK	2.81	2.24-3.52	2.28E-19
AURKB	2.19	1.81-2.66	1.60E- 15
CDC25A	2.44	1.98-3.01	5.5 l E-17
CENPE	2.5 1	2.12-2.98	3.47E-26
CKS2	2.9	2.44-3.44	2.38E-33
MAD2Ll	2.53	2.14-3	4.16E-27
MYBL2	2.27	1.87-2.77	3.80E-16
POLA2	3.29	2.59-4.17	l.47E-22
TK l	2 .08	1.75-2.48	2.28E-16
AURKA	3.12	2.54-3.84	6.57E-27
PTTGl	2 .74	2.23-3.36	1.2 l E-21
MCM2	3.11	2.48-3.89	4.34E-23
CCNB2	2.51	2.07-3.03	2.60E-2 1
PLKl	3.7	2.86-4.77	1.14E-23
TACC3	3.62	2.77-4.73	4.34E-21
RNASEH21	3.11	2.5-3.86	l .67E-24
BRCA l	2.38	1.95-2.9 1	1.09E-l 7
ESPLl	2.16	1.76-2.67	4.21E-13
KIF4A	3.19	2.55-3.98	1.69E-24
UBE2T	3.66	2.91-4.6	1.15E-28
ORC6	3.1	2.52-3.83	3.5 l E-26
UBE2S	2.71	2.28-3.21	2.25E-30
HELLS	3.43	2.72-4.34	7.10E-25
ASFl B	2.41	1.96-2.97	9.79E-17
SPC25	2.67	2.17-3.28	l.73E-20
GINSl	4.17	3.16-5.5	4.71E-24
DSCC l	3.24	2.6-4.05	3.84E-25
DCTPP l	3.48	2.8-4.33	4.28E-29
SPC24	2.43	2-2.95	2.58E-19

### Construction and validation of the E2F-associated risk model

**Figure 2:**
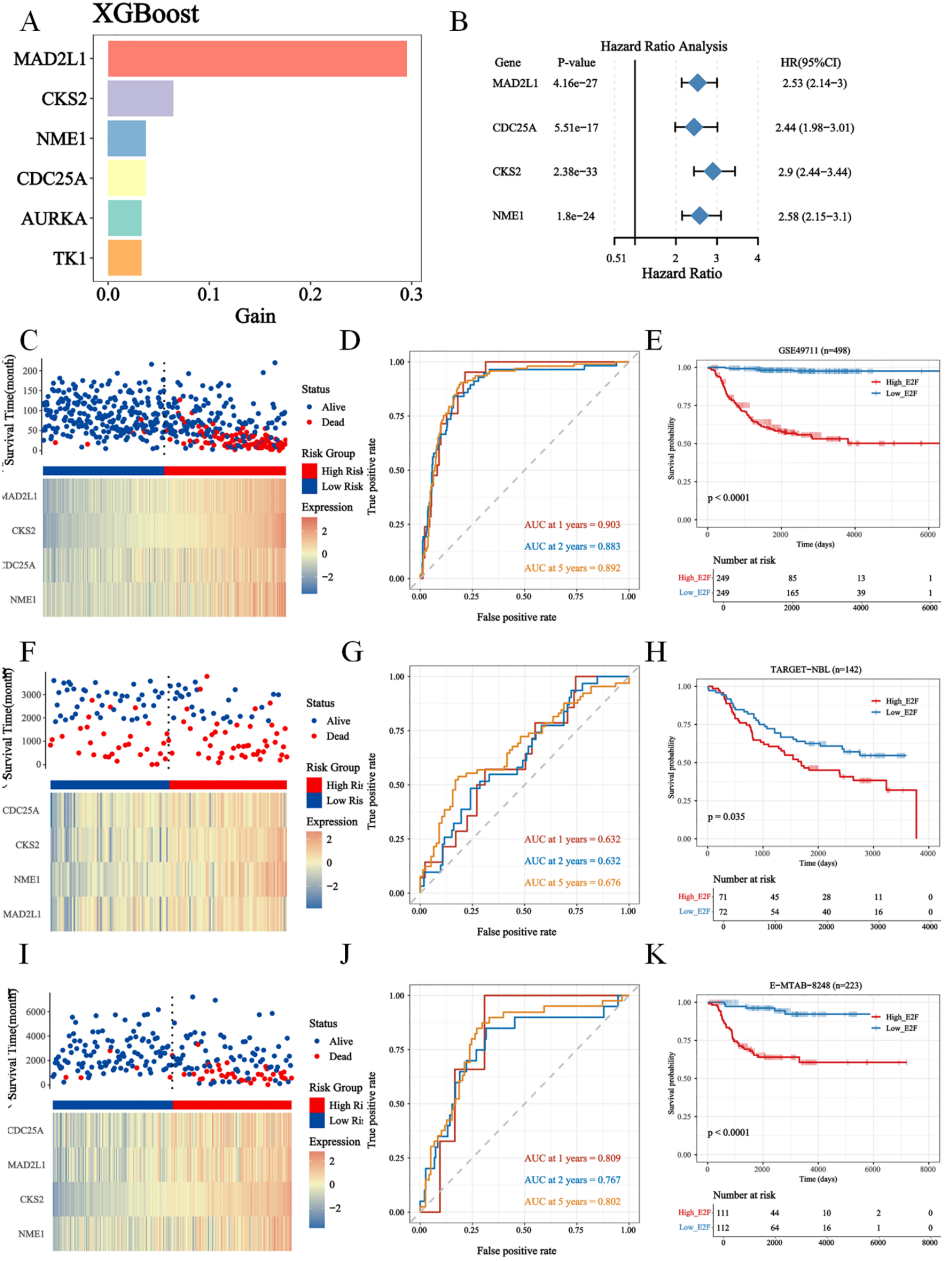
The screening of E2Fs gene in NB, and construction and validation of risk score model. **(A)** XGboost for E2Fs gene (*MAD2L1, CDC25A, CKS2, NME1*) scoring in NB (*P* < 0.05). **(B)** The cox analysis of E2Fs; **(C–E)** The Construction of GSE49711 training set. **(C)** Distribution of risk score and OS of GSE49711 training set; **(D)** Survival-dependent ROC curves validation at 1, 2, and 5 years of prognostic value of the prognostic index in GSE49711; **(E)** OS of GSE49711 cohort. **(F–H)** The Construction of TARGET-NBL validation set. **(F)** Distribution of risk score and OS of TARGET-NBL validation set; **(G)** Survival-dependent ROC curves validation at 1, 2, and 5 years of prognostic value of the prognostic index in TARGET-NBL; **(H)** OS of TARGET-NBLcohort. **(I–K)** The Construction of E-MTAB-8248 validation set. **(I)** Distribution of risk score and OS of E-MTAB-8248 validation set; **(J)** Survival-dependent ROC curves validation at 1, 2, and 5 years of prognostic value of the prognostic index in E-MTAB-8248; **(K)** OS of E-MTAB-8248 cohort.

Four genes were subsequently selected via XGBoost and least absolute shrinkage and selection operator (LASSO) regression to construct a prognostic signature aimed at classifying NB patients into two groups with different overall survival (OS) rates: the high-risk group and the low-risk group (**Figures 2A, B**, **Supplementary Figures S1C, D**). All patients were categorized into either the high-risk group or the low-risk group based on the median risk score. According to Kaplan–Meier analysis (**Figures 2C–K**), the OS of high-risk patients was significantly lower than that of low-risk patients.

In addition, the 1-, 2-, and 5-year OS rates based on area under the curve (AUC) values in the GSE49711 cohort, the TARGET-NBL cohort, and the E-MTAB-8248 cohort are shown in **Figures 2D, G, J**. To gain insight into the independent prognostic value of these 4 signature genes in the risk model, we performed univariate Cox regression analyses and found that 4 of them were detrimental to NB patients (**Figure 2B**). We plotted Kaplan–Meier survival curves to assess the prognostic value of each of the characterized genes, and the results were consistent with those of the univariate Cox regression analysis (**Supplementary Figure S2**).

### Prognostic validation of the four signature genes and construction of a nomogram with clinical characteristics

**Figure 3:**
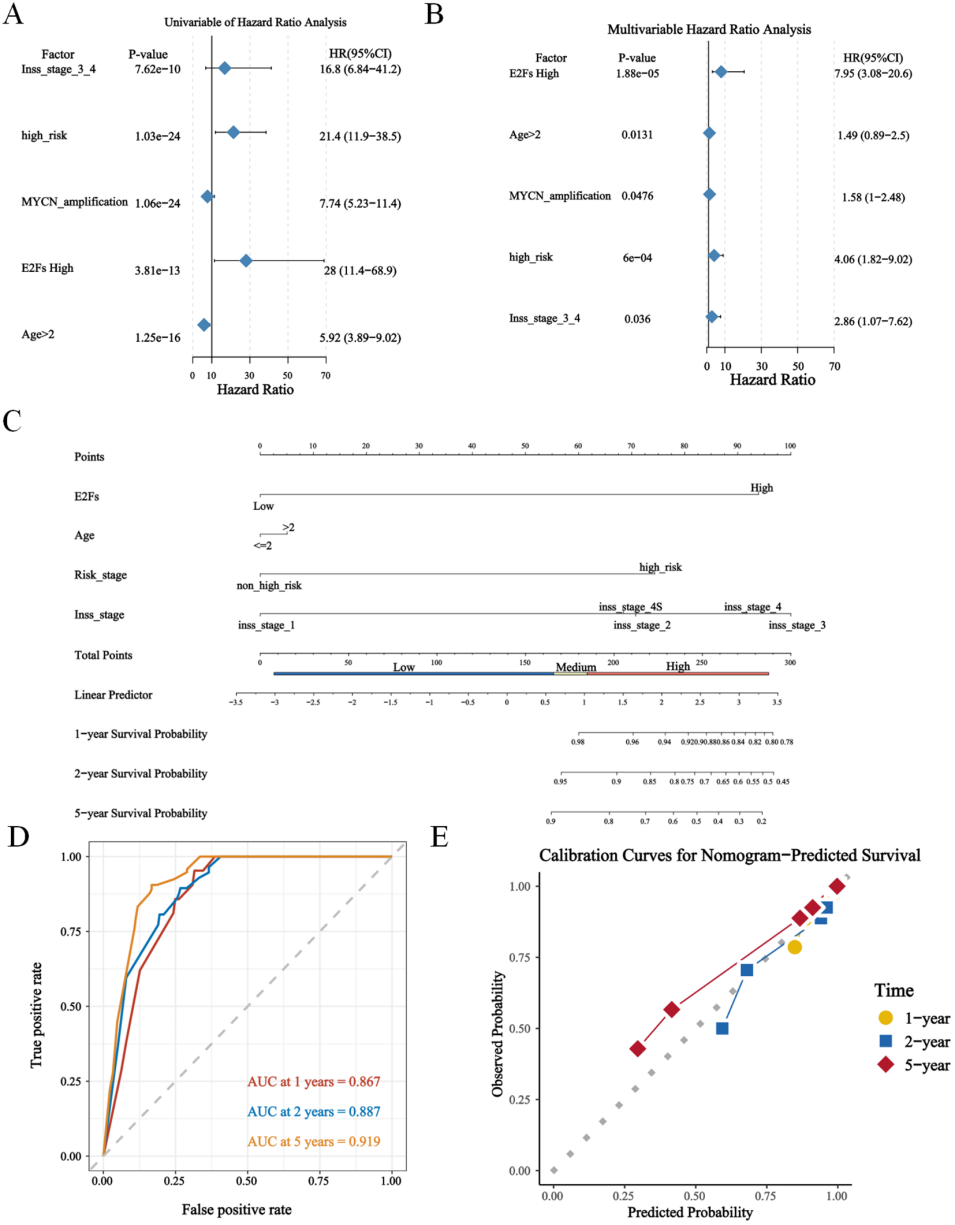
The construction of nomogram based on GSE49711. **(A, B)** The uni- and multi- cox analysis of nomogram factors were presented in forest plots (*P* < 0.05); **(C)** The nomogram plot; **(D)** The timeROC curve and calibration of nomogram, predicting 1-, 2-, and 5-year overall survival (OS) probabilities. **(E)** The calibration plot for the 5-year overall survival prediction using the constructed nomogram.

To investigate whether our risk model was associated with the clinical characteristics of NB, we performed a Wilcoxon rank sum test and found that the high-risk group had more advanced INSS staging and higher risk stratification (**Supplementary Figure S3**). Considering the different prognosis-related clinical characteristics between the two risk groups, we further investigated whether the risk model had similar or better predictive validity than other NB-independent prognostic factors did (**Supplementary Figure S3**). Stratified survival analysis revealed that the prognostic value of the E2F signature remained significant within the non-amplified subgroups (**Supplementary Figure S3E**), suggesting that the E2F signature provides independent and complementary prognostic information. Further mechanistic studies elucidated the interplay between MYCN genesets (*ARMC6, DCTPP1, EIF4G1, ELOVL6, FBL, HSPE1* and *PRMT1*) and E2F-related pathways (**Supplementary Figure S3F**). These findings suggest that MYCN may enhance E2F pathway activation, which could contribute to tumor aggressiveness and therapy resistance. In addition, this study included age, risk stratification, the INSS, the risk score and MYCN status in the Cox single multifactorial analysis (**Figures 3A, B**), and the ROC curves were evaluated for the predictive efficacy of MYCN status and risk stratification. The results suggested that risk stratification had better predictive efficacy (**Supplementary Figure S4A**). Therefore, this study constructed a column–line diagram to predict OS in patients, including three independent prognostic factors, namely, age, risk stratification, the INSS and the risk score (**Figure 3C**). The calibration plots indicated that the column-line plot may accurately estimate mortality (**Figures 3D, E**). The AUCs of the column-line plots were 0.867, 0.887, and 0.919 for 1-, 2-, and 5-year OS, respectively. Survival analyses suggested that the group with high model scores had a worse prognosis (**Supplementary Figure S4B**). The model was also further validated with the TARGET-NBL cohort and the E-MTAB-8248 cohort (**Supplementary Figures S4C–F**). The above results suggest that the risk model can be used either as an independent prognostic factor or in combination with existing clinical indicators.

### Enrichment analysis of proliferation gene sets in the risk score model

**Figure 4:**
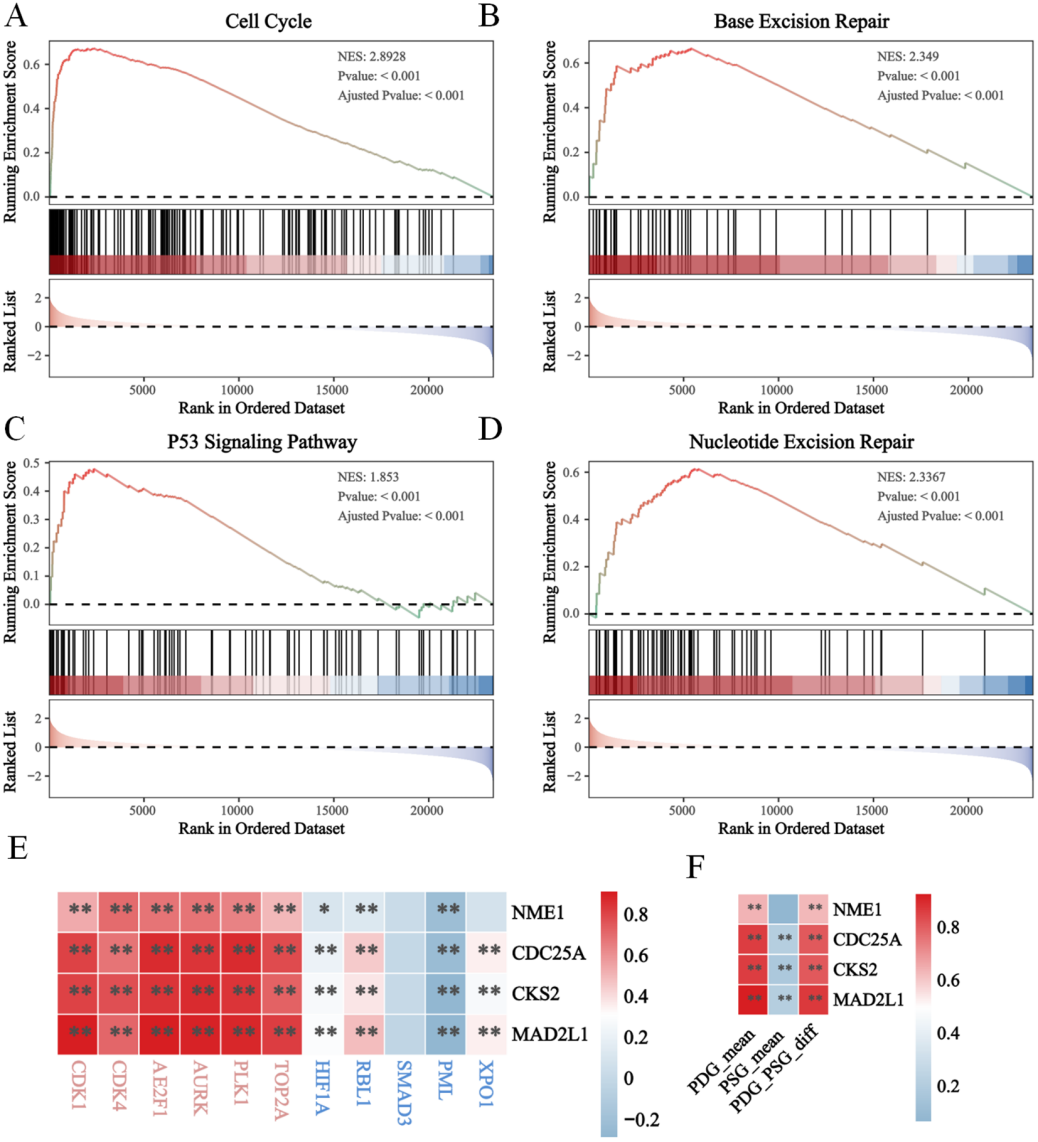
The enhanced proliferation in high E2Fs group of NB. The GSEA results revealled the activation of **(A)** cell cycle; **(B)** base excision; **(C)** p53 signaling pathway; **(D)** Nucleotide excision repair; **(E, F)** The heatmap of correlation between E2Fs genes and PDGs/PSGs in NB (*P* < 0.05). *P < 0.05, **P < 0.01.

To further validate the function of the risk model in proliferation, we performed GSEA pathway enrichment analysis and found that several proliferation-associated gene sets were enriched in the high-E2F group. Specifically, the gene sets related to the cell cycle (**Figure 4A**), base excision repair (**Figure 4B**), the P53 signaling pathway (**Figure 4C**), and nucleotide excision repair (**Figure 4D**) were significantly enriched. The normalized enrichment scores (NESs) for these pathways were 2.8928, 2.349, 1.853, and 2.2367 (*P*<0.05), indicating a strong association with high E2F expression. In addition, we found that the expression levels of the four E2F genes (*MAD2L1, CDC25A, CKS2, NME1*) were positively correlated with canonical proliferation-driving genes (PDGs), while showing a negative correlation with proliferation-suppressing genes (PSGs). These relationships suggest that elevated E2F activity contributes to enhanced proliferative capacity in neuroblastoma cells by simultaneously activating cell cycle progression and repressing cell cycle inhibitors. Together, these findings provide strong support for the role of the E2F signature in driving proliferation at the molecular level.

**Figure 5:**
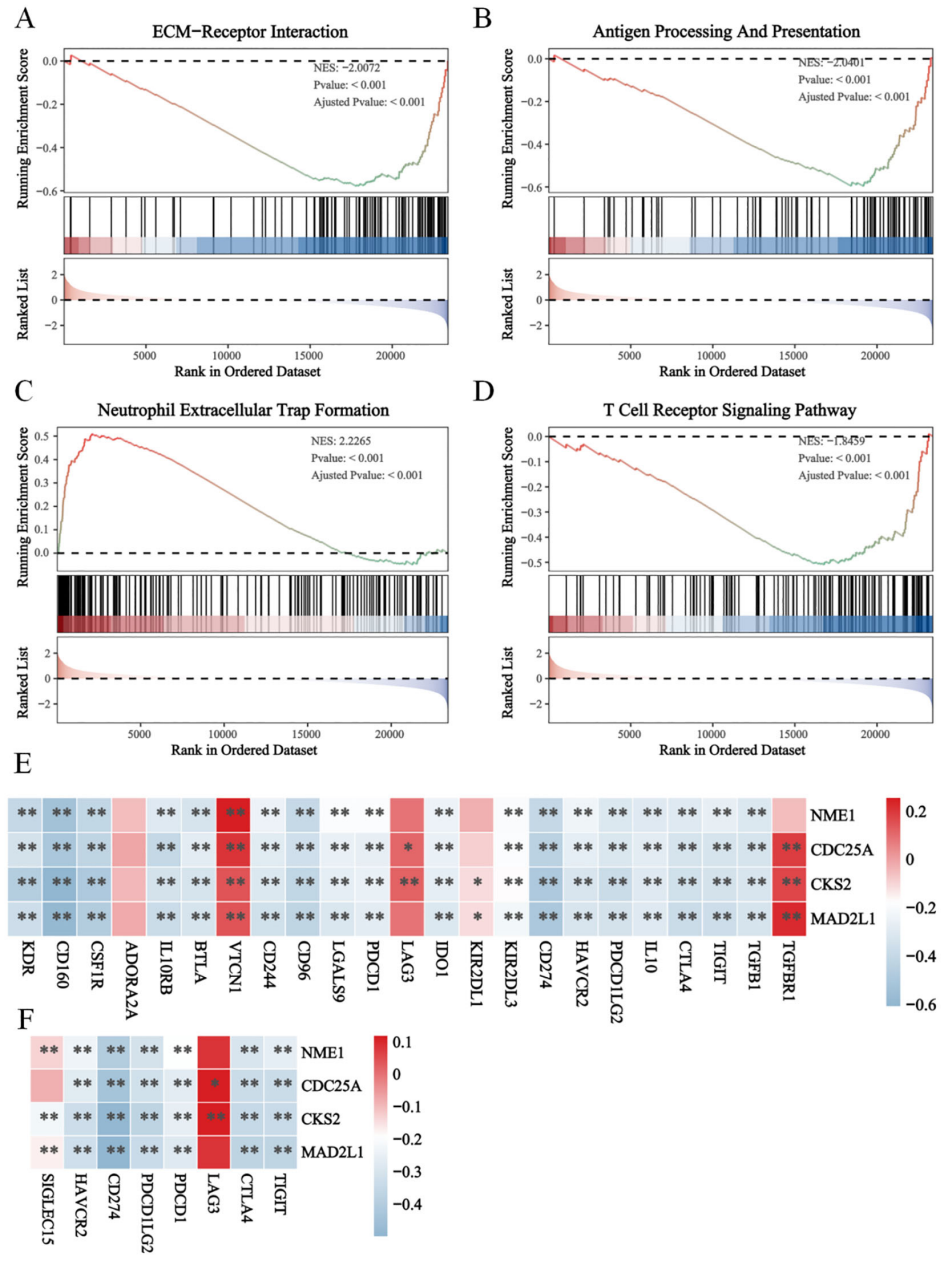
The immune suppression in high E2Fs-group NB. The GSEA results revealed the functional enrichment of **(A)** ECM-receptor interaction; **(B)** Antigen processing and presentation; **(C)** Neutrophil extracellular trap formation **(D)** T cell receptor signaling pathway; **(E)** The heatmap of correlations between immune suppression genes and E2Fs genes; **(F)** The heatmap of correlation between E2Fs and immune checkpoint (IC) in NB (*P* < 0.05). *P < 0.05, **P < 0.01.

Furthermore, we analyzed the correlation between E2Fs and immune suppression (**Figure 5E**) and between E2Fs and immune checkpoint (IC) genes in NB (**Figure 5F**). The heatmaps revealed significant correlations between several E2Fs and immune checkpoint genes, as well as between E2Fs and immune suppression, indicating potential interactions between these genesets. The enrichment analysis of immune genesets in the risk score model revealed significant associations with high E2F expression, providing further evidence for the functional role of the risk model in immunochemotherapy.

### Immune features of the high- and low-E2F groups

**Figure 6:**
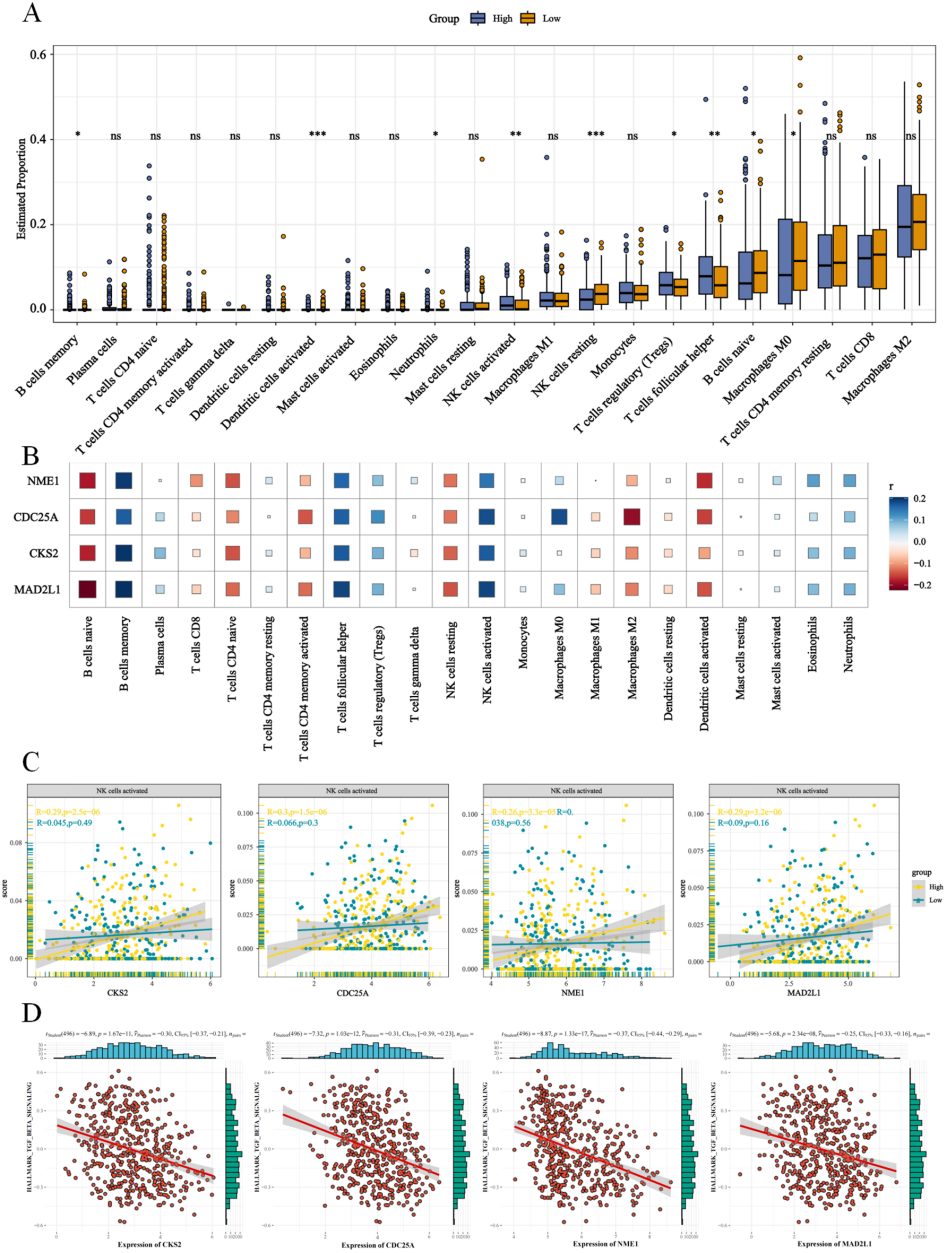
E2Fs are associated immune regulation in NB. **(A)** The proportion of 22 immune cells between high and low E2Fs group of NB (*P* < 0.001); **(B)** The heatmap of correlation between E2Fs and 22 immune cells in NB; **(C)** The enhanced correlations between E2Fs and NK cells activated; **(D)** The positive correlations between E2Fs genes and TGF-β signaling pathway (*P* < 0.05).

To investigate the tumor immune microenvironment (TIME) in different E2F risk groups, the infiltration levels of 22 immune cell types were compared (**Figure 6A**). Significant differences were observed in seven immune cell types. The low-E2F group exhibited increased infiltration of M0 macrophages, naïve B cells, resting NK cells, and activated dendritic cells. Conversely, the high-E2F group presented significantly elevated levels of memory B cells, neutrophils, follicular helper T cells, regulatory T cells (Tregs) and activated NK cells (**Figures 6A, B**). To further explore the association between E2F-related genes and immune modulation, we performed immune infiltration analysis with a focus on natural killer (NK) cells. Notably, high E2Fs riskscore showed a significant positive correlation with increased infiltration of NK cells. Mechanistically, pathway enrichment analysis suggested that this relationship may be partially mediated by the suppression of Transforming Growth Factor-β (TGF-β) signaling, a known inhibitor of NK cell activation. Specifically, samples with high E2F expression exhibited reduced TGF-β pathway activity scores and elevated NK cell cytotoxic signatures (**Figure 6D**), suggesting that these genes may play a role in influencing NK cell activity within the TME.

Furthermore, GSEA of the E2F groups revealed immune suppression mechanisms, including the downregulation of immune checkpoints, T/B-cell receptor signaling, and antigen presentation pathways (**Figures 5A–D**). The correlation heatmap demonstrated a negative association between E2F expression and immune checkpoint-related genes (**Figure 5E**), further supporting the hypothesis of immune response suppression. These trends were also observed in the pancancer analysis (**Supplementary Figure S6**). Given these findings, it can be speculated that the improved overall survival (OS) of patients in the low-E2F group may be partially attributed to the increased infiltration of immune cells with antitumor activity.

### Tumor mutation burden between E2F groups in NB

**Figure 7:**
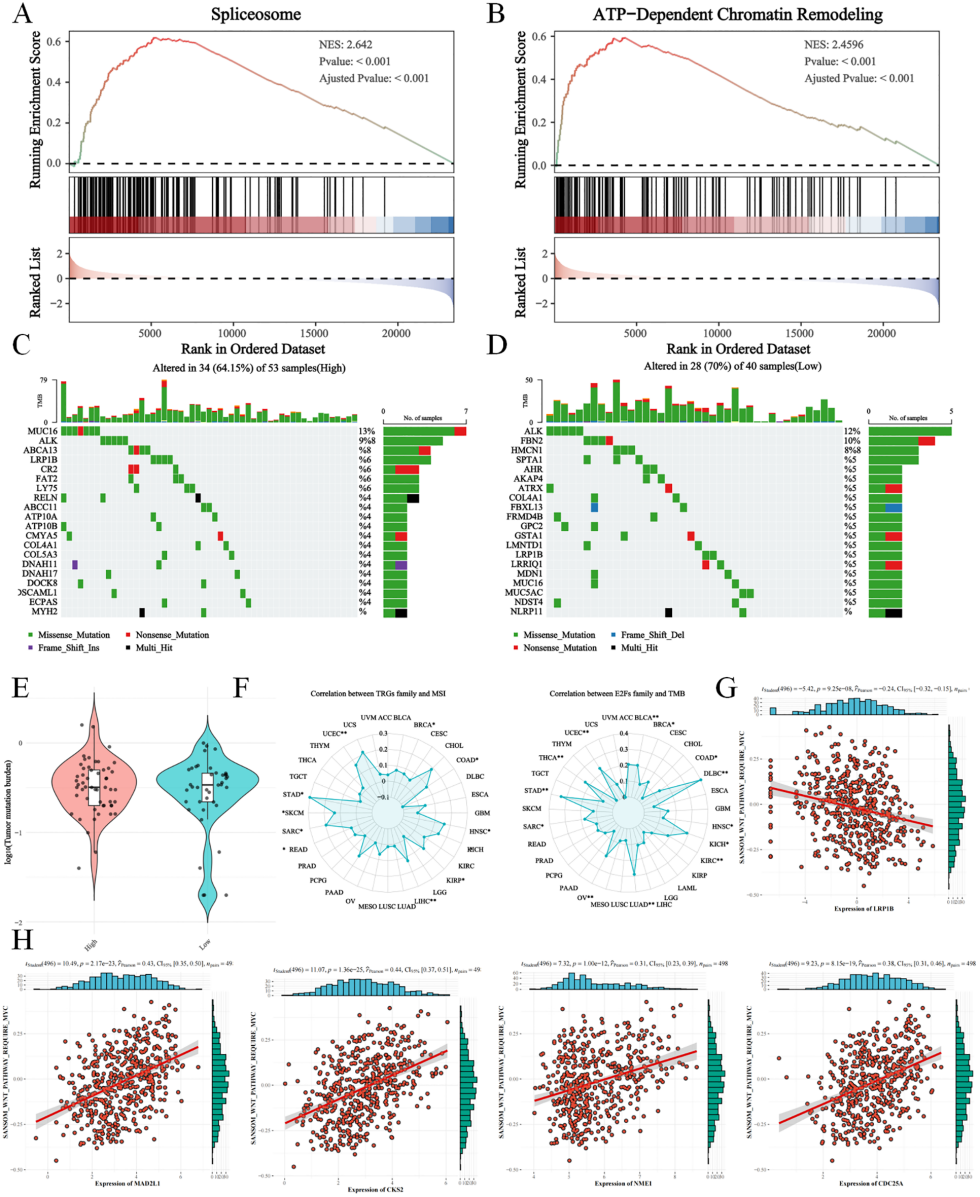
The tumor mutation burden (TMB) analysis between E2Fs-group NB **(A)** The activation of spliceosome in GSEA analysis; **(B)** The activation of ATP-dependent chromatin remodeling in GSEA analysis; (C&D) The TMB in high E2Fs NB and low E2Fs NB; **(E)** The comparison of TMB between low E2Fs and high E2Fs NB; **(F)** The correlations of E2Fs with microsatellite instability (MSI) and TMB in pan-cancer; **(G)** The correlations of LRP1B with WNT signaling pathway; **(H)** The correlations of E2Fs genes with WNT signaling pathway (*P* < 0.05).

NB in the high-E2F group presented increased activity in the spliceosome, ATP-dependent chromatin remodeling, and other pathways related to genome regulation (**Figures 7A, B**). To analyze the tumor mutation burden (TMB) during NB progression, simple nucleotide variations (SNVs) were compared between the E2F groups (**Figures 7C, D**). No significant differences in overall TMB were observed (**Figure 7E**). However, the high E2F group presented increased frequencies of *ABCA13*, *LRP1B*, and *ATP10B* missense/nonsense mutations, suggesting a potential link between E2Fs and tumor-suppressor gene alterations. These findings were further validated via microsatellite instability (MSI) and TMB pancancer analyses, indicating that E2Fs may influence TMB through mechanisms related to cell proliferation and epigenetic modification (**Figure 7F**).

We observed that both ABCA13 and LRP1B are expressed at higher levels in MYCN non-amplified neuroblastoma, a subgroup typically associated with less heterogeneous outcomes. Notably, lower expression of either gene correlates with significantly worse prognosis (**Supplementary Figure S6**). Using ssGSEA, we found that E2F family gene expression is positively correlated with WNT signaling activity, whereas *LRP1B* expression shows a negative correlation with WNT signaling (**Figures 7G, H**). These findings suggest that *LRP1B* may act as a suppressor of WNT-driven oncogenic processes, potentially linking its loss-of-function mutations to pathway dysregulation and tumor progression.

### Sensitivity prediction analysis of common drugs

**Figure 8:**
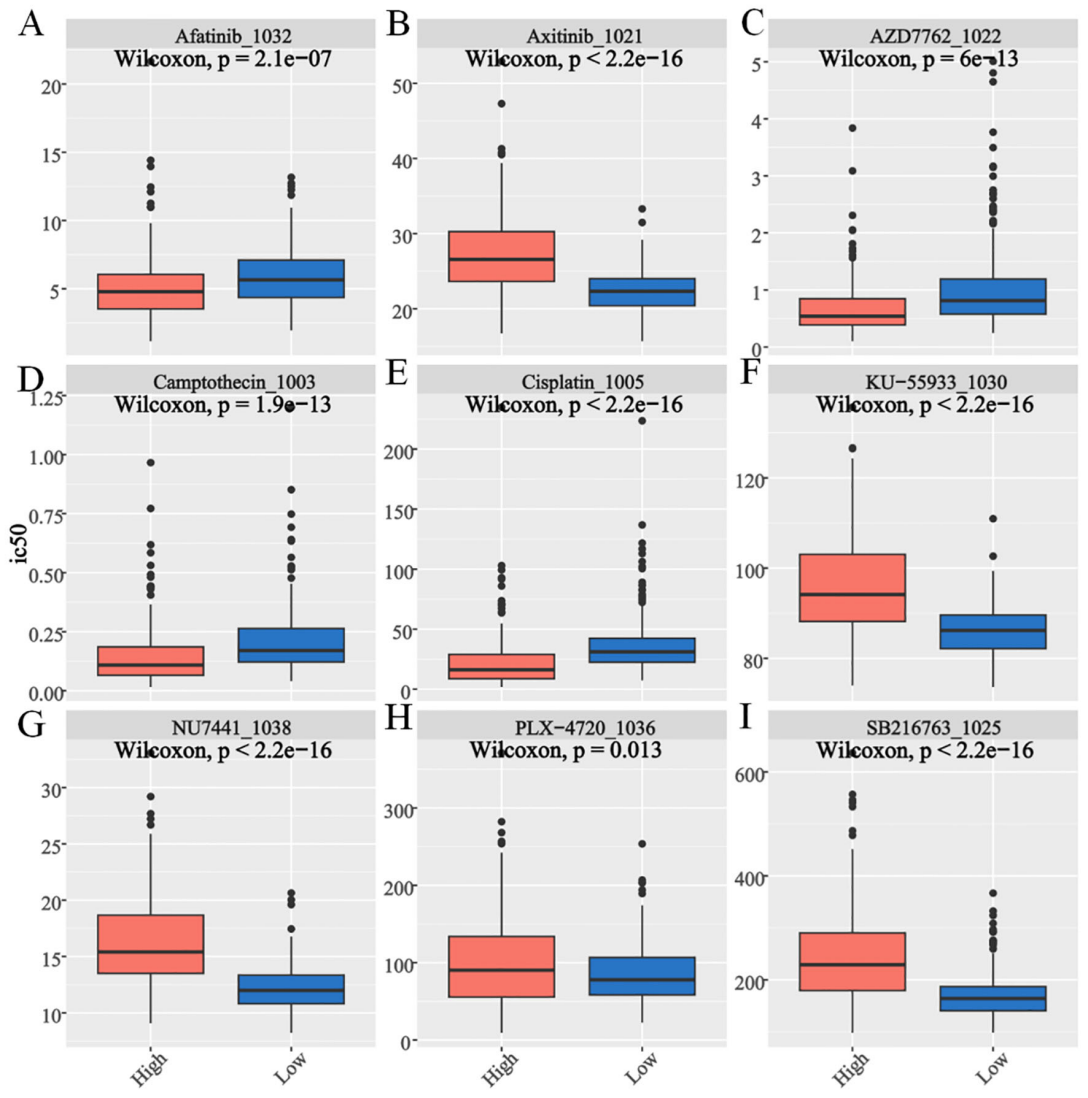
Drug sensitivity assessment for E2Fs-associated nomogram evaluation of GSE49711 cohort. The box plot shows the **(A)** Afatinib. **(B)** Axitinib. **(C)** AZD7762. **(D)** Camptothecin. **(E)** Cisplatin. **(F)** KU-55933. **(G)** NU7441. **(H)** PLX-4720. **(I)** SB216763 (*P* < 0.05).

The Wilcoxon test was conducted to assess differences in drug sensitivity (IC50 values) between the two scoring groups. Significant variations were observed for 197 drugs, including afatinib, AZD7762, camptothecin, and cisplatin. Notably, the high-scoring group exhibited greater sensitivity to axitinib, KU-55933, NU7441, PLX-4720, and SB216763 than the low-scoring group did (P < 0.05, **Figures 8A–I**). These findings underscore the potential of risk stratification in guiding personalized therapeutic strategies to enhance treatment efficacy and patient outcomes.

## Discussion

High E2F activity is strongly associated with high-risk neuroblastoma (NB), as evidenced by its correlation with aggressive tumor phenotypes, immune suppression, and altered tumor mutation burden (TMB). The E2F transcription factor family plays a critical role in cell cycle regulation and tumor progression, and its dysregulation contributes to NB malignancy and poor prognosis. This study highlight the potential of the E2F signature as a clinically actionable prognostic tool in neuroblastoma, which is consistent with previous studies linking E2F-mediated cell cycle dysregulation to high-risk NB (11, 12). Beyond its robust association with poor outcomes, the E2F signature could be implemented as a complementary biomarker to refine existing risk stratification systems, such as MYCN amplification and INSS staging. Notably, E2F1, a key regulator of the G1/S transition, is significantly upregulated in high-risk NB, particularly in patients with MYCN amplification, a well-established hallmark of aggressive NB (13). Additionally, E2F3 and E2F7, which are both involved in promoting tumor proliferation and inhibiting apoptosis, exhibited increased expression in high-risk NB, further emphasizing their role in tumor progression (14).

GSEA revealed that high-E2F neuroblastomas are significantly enriched in proliferative pathways, including cell cycle regulation (NES=2.89, p<0.001), DNA repair mechanisms (base/nucleotide excision repair: NES=2.35/2.24), and p53 signaling (NES=1.85) (**Figures 4A–D**), which is consistent with the established roles of E2Fs in driving cell cycle progression through direct transcriptional activation of cyclins and CDKs (15). The concomitant activation of DNA repair pathways likely represents a compensatory response to replication stress induced by E2F-mediated hyperproliferation, as demonstrated in other E2F-dysregulated cancers (16). Importantly, this proliferative phenotype was associated with broad immunosuppressive features, including (1) significant downregulation of PD-L1/CTLA-4 (**Figure 6E**) and impaired antigen presentation (**Figures 6A–D**), mirroring the immune-evasion mechanisms observed in MYC-driven tumors where cell cycle regulators directly suppress interferon signaling (17); (2) reduced T/B-cell receptor signaling consistent with E2F1-mediated suppression of T-cell activation genes (18); and (3) paradoxical NK cell activation without effector function (**Figure 5C**), resembling the dysfunctional NK populations in E2F3-overexpressing pediatric tumors (19). These findings collectively position E2F hyperactivity as a dual driver of proliferative advantage and immune evasion in high-risk neuroblastoma, suggesting that therapeutic strategies combining CDK4/6 inhibitors (to target E2F activation) with NK cell engagers or TIM-3 blockade (to overcome immune suppression) may be particularly effective, as recently demonstrated in preclinical models of E2F3-amplified neuroblastoma (20).

Immune cell infiltration analysis revealed distinct immunophenotypes between the E2F expression groups, underscoring their role in shaping the tumor microenvironment (TME). The low-E2F group exhibited greater infiltration of M0 macrophages, naïve B cells, resting NK cells, and activated dendritic cells (**Figures 5A, B**), suggesting a more immunogenic TME, as dendritic cell activation is critical for antigen presentation and T-cell priming (21), whereas naïve B cells may contribute to tertiary lymphoid structure formation, which is associated with favorable outcomes (22). In contrast, the high-E2F group displayed elevated populations of immunosuppressive cells, including memory B cells, neutrophils, follicular helper T cells (Tfhs), regulatory T cells (Tregs), and activated NK cells. The enrichment of Tregs and neutrophils aligns with established immune evasion mechanisms in neuroblastoma (NB), where Tregs suppress antitumor immunity (23) and neutrophils promote protumorigenic inflammation (24). Notably, E2F-associated genes (CKS2, CDC25A, NME1, and MAD2L1) were positively correlated with activated NK cell infiltration (**Figure 5C**), suggesting cell cycle-related immune modulation, which is consistent with reports linking proliferative signaling to NK cell synapse regulation (25). However, the high-E2F group also exhibited downregulation of immune checkpoints (**Figure 6E**) and impaired T/B-cell receptor pathways (**Figures 6A–D**), indicative of systemic immunosuppression, akin to MYC-driven immune escape (17). These findings collectively implicate E2Fs in fostering an immune-suppressive TME, where the survival advantage of low-E2F syndrome patients may reflect preserved immune surveillance, whereas high-E2F syndrome activity facilitates immune evasion, suggesting therapeutic potential for combining E2F pathway inhibition with immunotherapy.

Correlation analysis revealed significantly reduced immune checkpoint expression in high-E2F tumors (**Figure 5E**), which may explain the limited efficacy of immune checkpoint inhibitors (ICIs) in neuroblastoma, as observed in other cancers where low checkpoint expression correlates with poor immunotherapy response (26). Interestingly, while not statistically significant, we noted a trend toward higher stromal scores in low-E2F tumors, potentially reflecting cancer-associated fibroblast (CAF)-mediated immune checkpoint induction of T cells (27), a mechanism that could sustain immunogenicity in these tumors. This dichotomy suggests that low-E2F patients, with preserved checkpoint expression and immune infiltration, may derive greater benefit from ICIs, whereas high-E2F patients likely require combinatorial strategies targeting both the cell cycle and alternative immune pathways. Preclinical evidence supports this approach, demonstrating synergy between E2F inhibition and PD-1 blockade in MYC-driven tumors (17), suggesting that our E2F-immune classifier could guide personalized immunotherapeutic strategies in NB beyond conventional risk stratification.

Moreover, our findings highlight a potential mechanism by which E2F-related genes enhance NK cell infiltration in the tumor microenvironment. Previous studies have shown that TGF-β signaling suppresses NK cell function by inhibiting cytotoxicity and proliferation (28). In this study, we observed that high-E2F groups displayed both decreased TGF-β pathway activity and increased NK cell infiltration, suggesting that E2Fs may indirectly promote NK cell activation by repressing TGF-β signaling. This aligns with emerging evidence linking E2F transcription factors to immune regulation beyond cell cycle control. These insights suggest a dual role for E2Fs in tumor progression and immune modulation, potentially offering novel therapeutic opportunities for enhancing anti-tumor immunity in neuroblastoma.

Tumor mutation burden (TMB) analysis revealed no significant differences in overall mutation rates between the E2F expression groups, suggesting that E2F dysregulation does not globally increase genomic instability in neuroblastoma. However, the high-E2F group presented an increased frequency of ABCA13, LRP1B, and ATP10B mutations, which may reflect E2F-mediated transcriptional regulation of DNA repair pathways and increased replication stress in proliferating cells. Specifically, E2F transcription factors directly regulate DNA repair genes and chromatin remodelers, potentially creating a permissive environment for specific mutation accumulation (29). The observed LRP1B mutations, which are located in a genomic region vulnerable to replication stress (30), may confer therapeutic vulnerability to PARP inhibitors (31). Similarly, ABCA13 and ATP10B mutations can alter membrane lipid composition, potentially increasing susceptibility to lipid-based chemotherapeutics (32). These findings align with those of pancancer analyses showing that E2F-high tumors present characteristic mutation signatures associated with replication stress (33) and specific mutation patterns in chromatin regulators (34). While E2F expression does not directly influence overall TMB, it may contribute to neuroblastoma malignancy through these focal mutations and epigenetic modifications, supporting the link between E2F activity and high-risk disease.

Notably, our findings suggest a potential mechanistic link between LRP1B mutation and WNT signaling activation in neuroblastoma. Specifically, LRP1B expression was negatively correlated with WNT pathway activity based on ssGSEA analysis, indicating that loss or mutation of LRP1B may release inhibitory control over the WNT/β-catenin signaling cascade. This is consistent with previous studies in other cancer types, where LRP1B mutations have been associated with WNT pathway activation and subsequent tumor progression. Aberrant WNT signaling can enhance tumor cell proliferation, migration, and stemness, and may also promote immune evasion by remodeling the tumor microenvironment (35).

Drug sensitivity analysis revealed distinct therapeutic vulnerabilities between the E2F expression groups. The low-E2F group exhibited greater sensitivity to immune checkpoint inhibitors, aligning with their enriched immune activation signature, as previously reported in tumors with lower proliferative activity (33, 36). Further sensitivity prediction analysis, assessed by the Wilcoxon rank-sum test, revealed significant differences in drug response between the two groups. Notably, the high-E2F cohort displayed increased sensitivity to targeted agents, including axitinib (a VEGFR inhibitor), KU-55933 (an ATM kinase inhibitor), NU7441 (a DNA-PK inhibitor), PLX-4720 (a BRAF inhibitor), and SB216763 (a GSK-3β inhibitor) (P < 0.05). These findings are supported by prior studies linking E2F hyperactivity to dependency on DNA repair pathways (37) and growth factor signaling (38).

Collectively, these results underscore the potential of E2F-based stratification to guide personalized therapeutic strategies, optimizing drug selection for high-versus low-E2F tumors. Although clinical implementation would require further prospective validation and technical standardization, the signature’s reproducibility across independent cohorts underscored its feasibility for future use. Moreover, future studies would aim to validate the signature in larger, prospective, multicenter clinical cohorts to further assess its clinical utility and applicability across diverse patient populations.

While the E2F-based risk model integrates proliferative and immune characteristics to improve neuroblastoma stratification, several limitations should be noted. The retrospective nature of public database-derived data may not fully capture disease heterogeneity across populations, and the moderate sample size limits the statistical power for subtle associations. Additionally, bulk transcriptomics cannot resolve spatial heterogeneity in E2F activity and immune infiltration, a critical consideration given known regional microenvironment variations in neuroblastoma (26). Future studies should employ spatial transcriptomics and single-cell RNA sequencing to map E2F-immune interactions at subregion resolution, validate the model in multicenter prospective cohorts, investigate the regulatory roles of noncoding RNAs (39), and evaluate E2F-targeting combination therapies in preclinical models to advance clinical translation.

## Data Availability

The original contributions presented in the study are included in the article/**Supplementary Material**. Further inquiries can be directed to the corresponding authors.

## Supplementary material

Supplementary Figure 1 Screening for E2Fs-related genes in high-risk neuroblastoma. **(A)** Consensus values range from 0 to 1; **(B)** The corresponding relative change in area under the cumulative distribution function (CDF) curves when cluster number changes from k to k+1. The range of k changed from 2 to 9, and the optimal k = 2; **(C)** LASSO coefficient profiles; **(D)** Selection of the tuning parameter (lambda) in the LASSO model by 10-fold cross-validation based on minimum criteria for OS.

Supplementary Figure 2
**(A–D)** The K-M curve of the E2Fs-related genes (*MAD2L1、CDC25A、CKS2、NME1*) revealled the association between overexpression and poor prognosis (*P* < 0.05).

Supplementary Figure 3 The E2Fs expression in distinct **(A)** INSS stages; **(B)** *MYCN*-status; **(C)** risk stages; **(D)** Age; **(E)** The K-M curve of the E2Fs riskscore in NB patients of non-amplifed *MYCN*; **(F)** The heatmap of correlation between E2Fs genes and *MYCN* geneset (*ARMC6, DCTPP1, EIF4G1, ELOVL6, FBL, HSPE1 and PRMT1*) in NB (*P* < 0.05).

Supplementary Figure 4
**(A)** The ROC comparison of risk stage and *MYCN*-status; **(B)** The K-M curve of nomogram; **(C)** The timeROC and **(D)** calibration curves in TARGET-NBL; **(E)** The timeROC and **(F)** calibration curves in E-MTAB-8248.

Supplementary Figure 5
**(A)** The heatmap of correlation between TRGs and immune checkpoint in pan-cancer; **(B)** The heatmap of correlation between TRGs and immune suppression in pan-cancer.

Supplementary Figure 6
**(A)** The LRP1B expression in distinct MYCN status; **(B)** The K-M curve of the LRP1B expression in NB patients; **(C)** The ABCA13 expression in distinct MYCN status; **(D)** The K-M curve of the ABCA13 expression in NB patients (*P* < 0.05).
