# Supplementary material for: Precision prognostication in neuroblastomas via clinically validated E2F activity signatures
Source: Front Immunol. 2025 Jun 17;16:1612667. doi: 10.3389/fimmu.2025.1612667 (PMC12209246; doi:10.3389/fimmu.2025.1612667)
Supplement: Supplementary Figure 1 — Screening for E2Fs-related genes in high-risk neuroblastoma. (A) Consensus values range from 0 to 1; (B) The corresponding relative change in area under the cumulative distribution function (CDF) curves when cluster number changes from k to k+1. The range of k changed from 2 to 9, and the optimal k = 2; (C) LASSO coefficient profiles; (D) Selection of the tuning parameter (lambda) in the LASSO model by 10-fold cross-validation based on minimum criteria for OS. [file DataSheet1.docx]

**Table S1 E2F-related geneset**

**Table S2 Nomogram clinical information**

**Table S3 Up-DEGs in HR-NB**

**Table S4 Veen genes**

**Table S5 E2F group in GSE49711**

**Table S6 E2F group in E-MTAB-8248**

**Table S7 E2F group in TARGET-NBL**

**
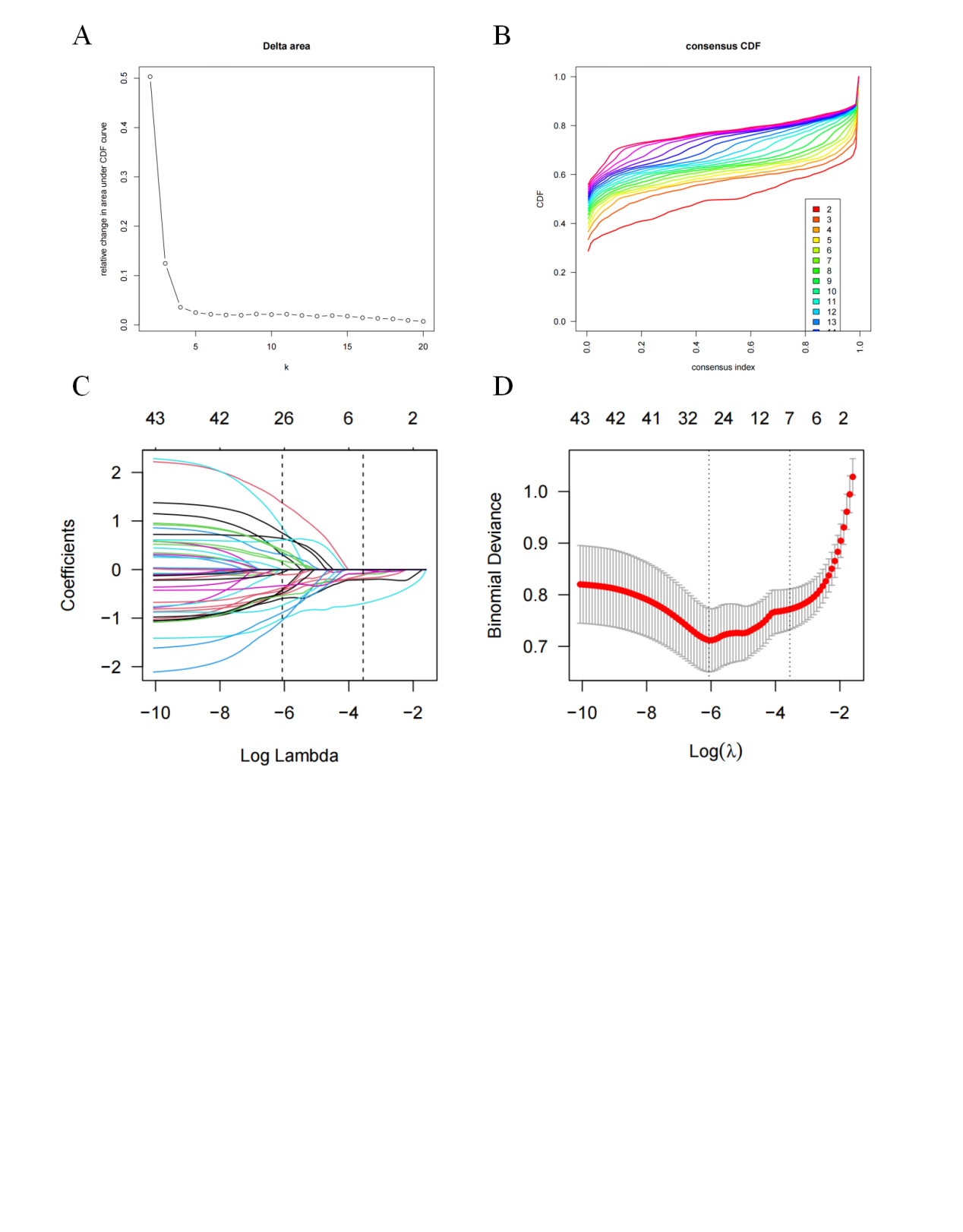
**

**Figure S1** Screening for E2Fs-related genes in high risk neuroblastoma. (A) Consensus values range from 0 to 1; (B) The corresponding relative change in area under the cumulative distribution function (CDF) curves when cluster number changes from k to k+1. The range of k changed from 2 to 9, and the optimal k = 2; (C) LASSO coefficient profiles; (D) Selection of the tuning parameter (lambda) in the LASSO model by 10-fold cross-validation based on minimum criteria for OS.

**
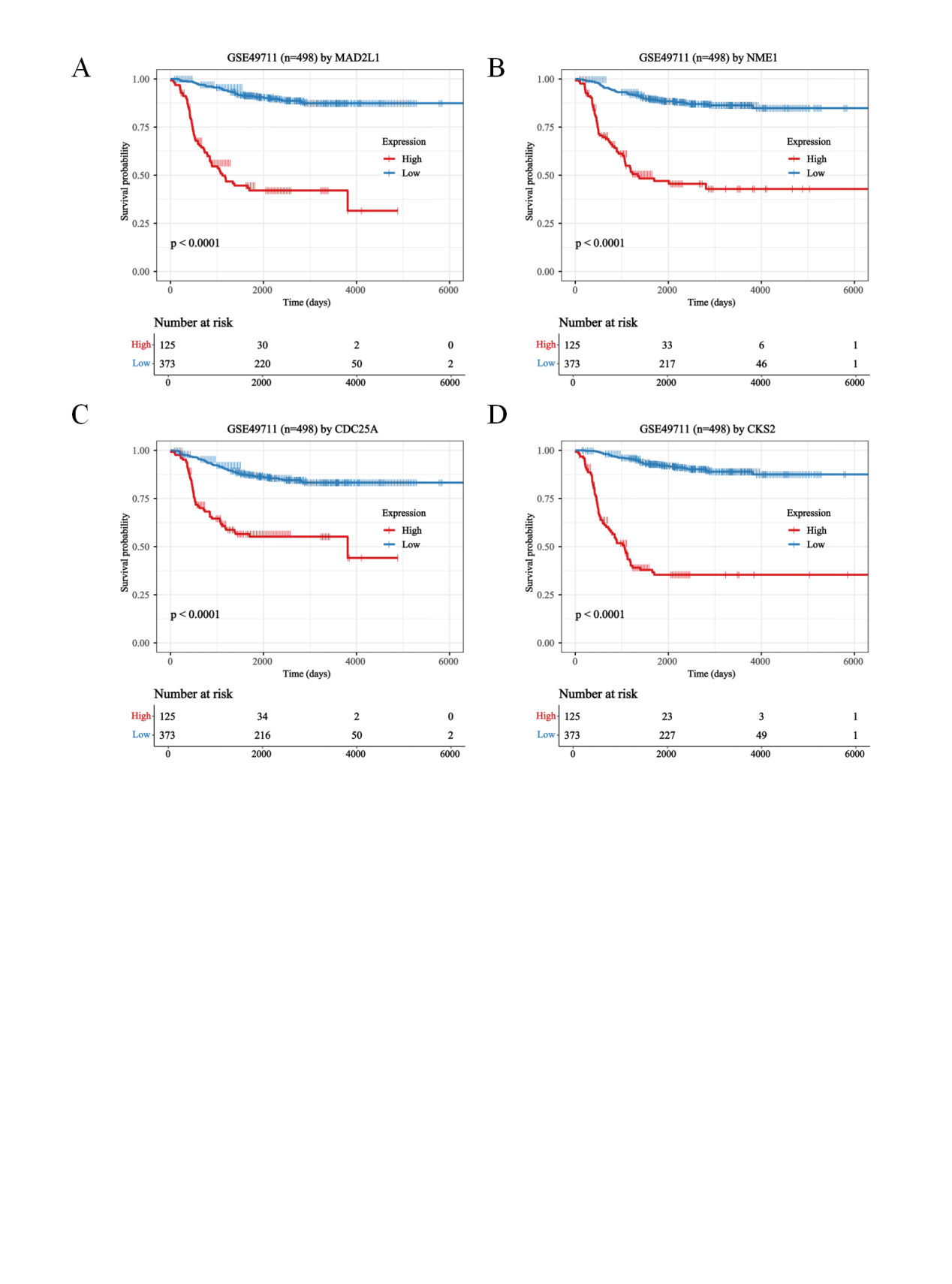
**

**Figure S2** (A-D)The K-M curve of the E2Fs-related genes (*MAD2L1、CDC25A、CKS2、NME1*) revealled the association between overexpression and poor prognosis (*P* < 0.05).


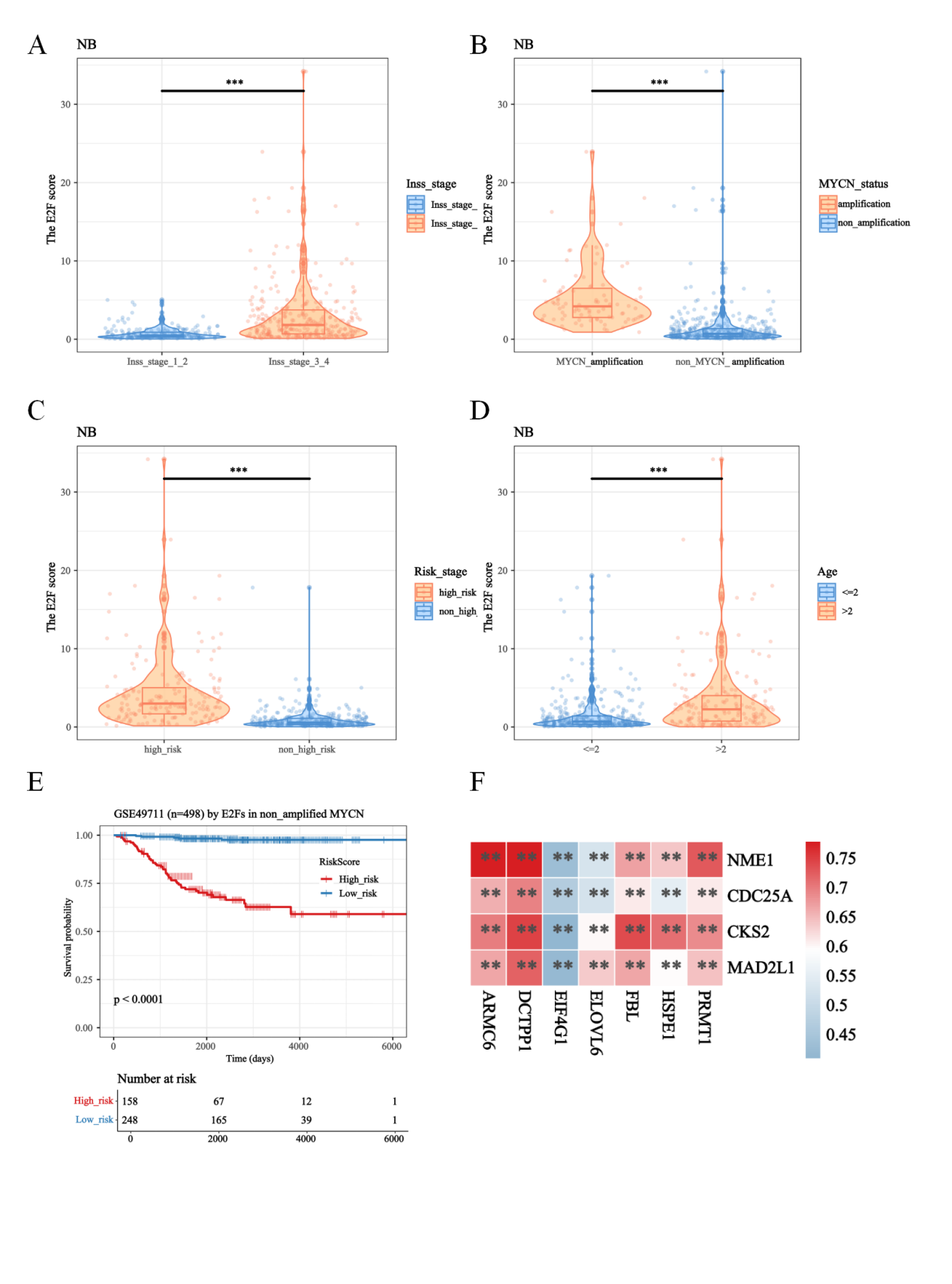


**Figure S3** The E2Fs expression in distinct (A) INSS stages; (B) *MYCN*-status; (C) risk stages; (D) Age; (E) The K-M curve of the E2Fs riskscore in NB patients of non-amplifed *MYCN*; (F) The heatmap of correlation between E2Fs genes and *MYCN* geneset (*ARMC6, DCTPP1, EIF4G1, ELOVL6, FBL, HSPE1 and PRMT1*) in NB (*P* < 0.05).

**
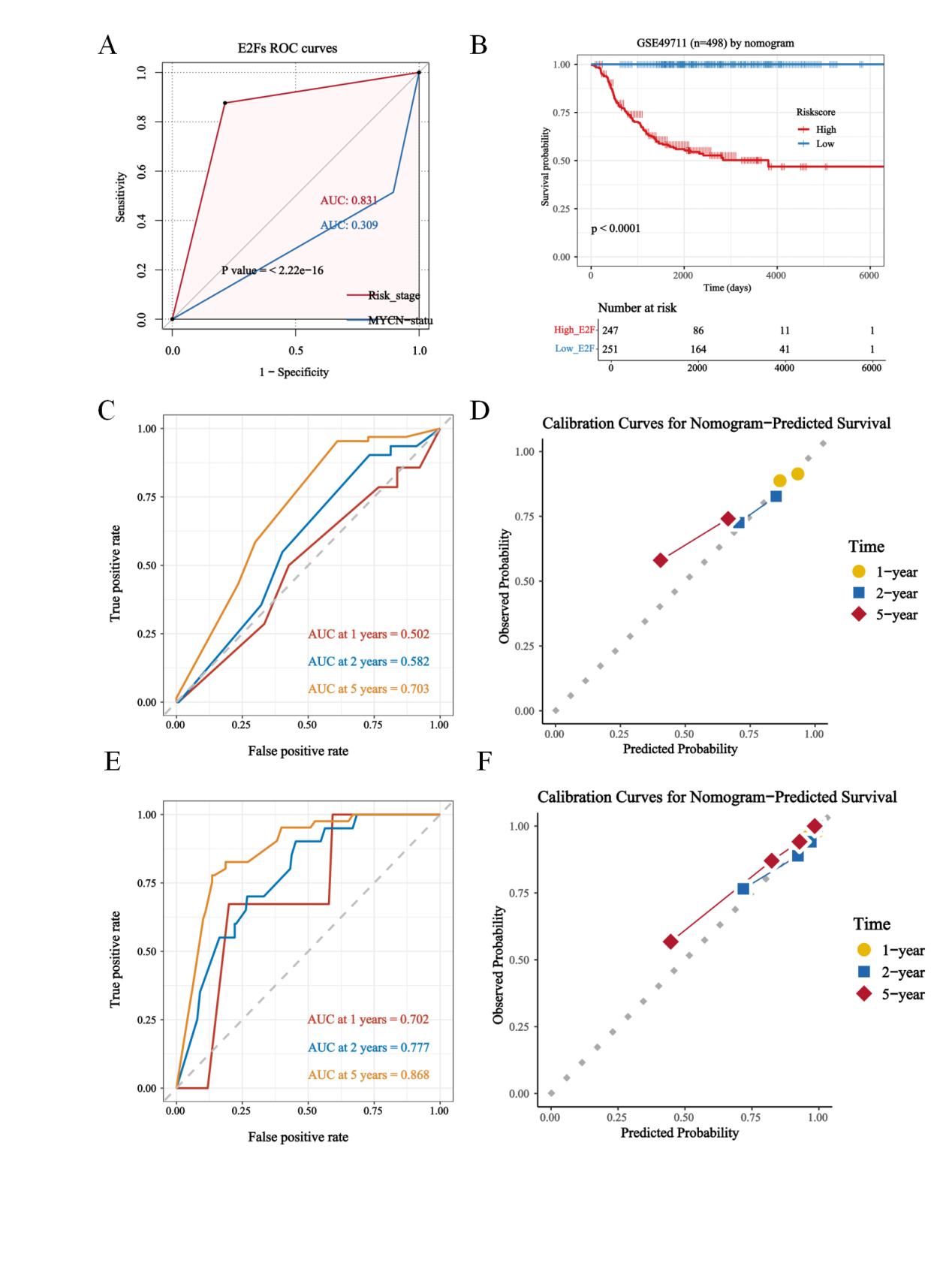
Figure S4** (A) The ROC comparison of risk stage and *MYCN*-status; (B) The K-M curve of nomogram; (C) The timeROC and (D) calibration curves in TARGET-NBL; (E) The timeROC and (F) calibration curves in E-MTAB-8248.


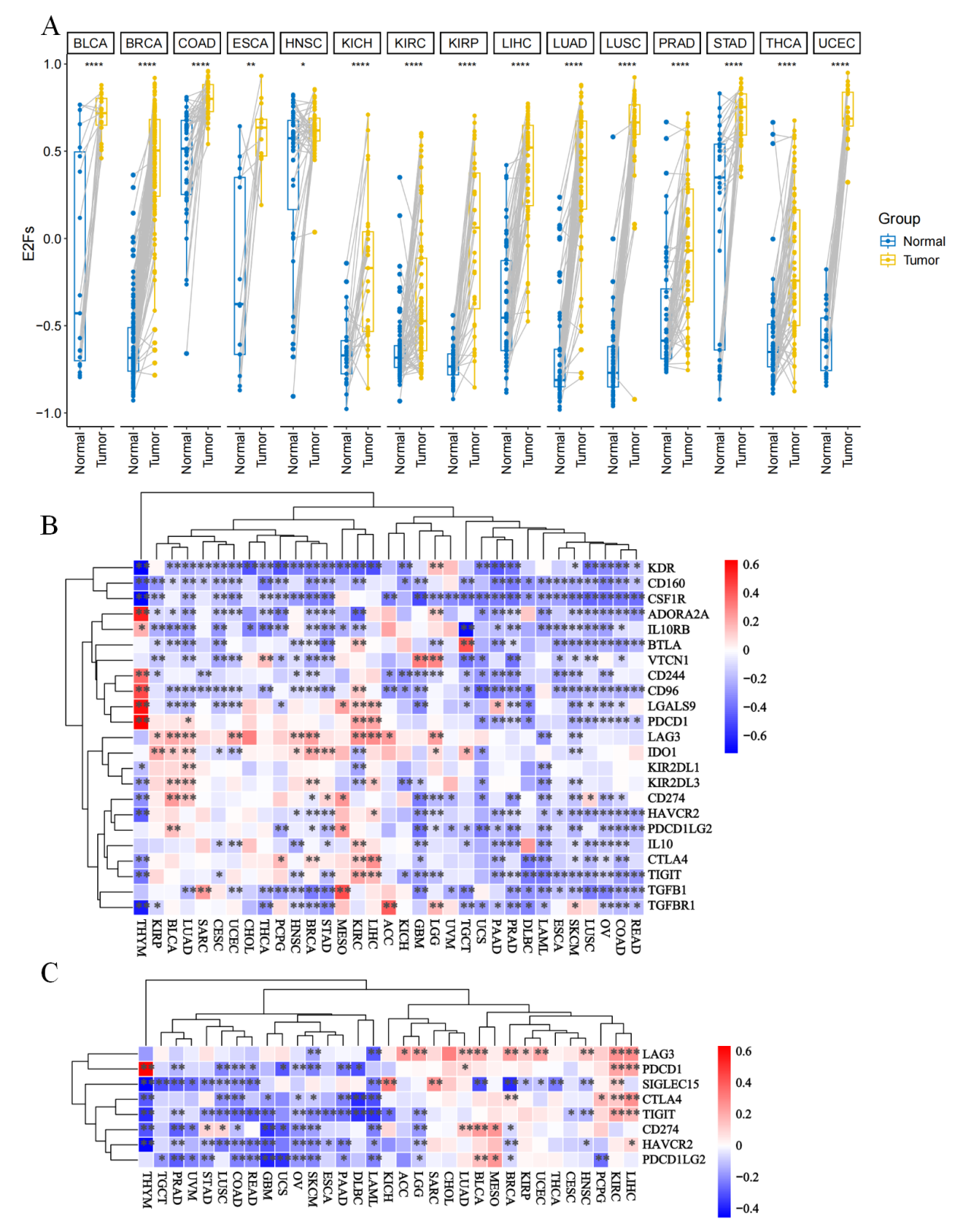


**Figure S5** (A) The heatmap of correlation between TRGs and immune checkpoint in pan-cancer; (B) The heatmap of correlation between TRGs and immune suppression in pan-cancer.


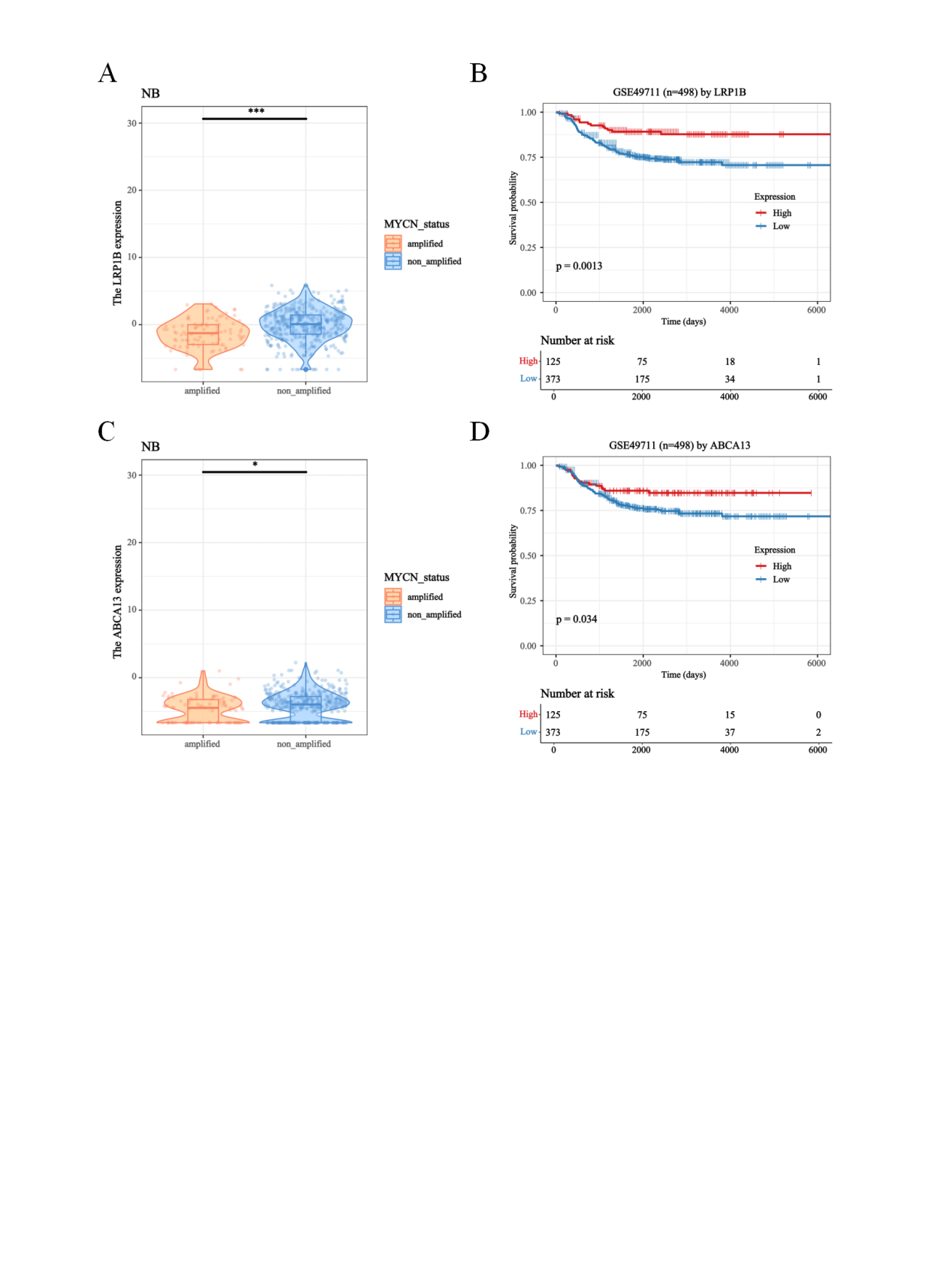


**Figure S6 (A)** The LRP1B expression in distinct MYCN status; (B) The K-M curve of the LRP1B expression in NB patients; (C) The ABCA13 expression in distinct MYCN status; (D) The K-M curve of the ABCA13 expression in NB patients (*P* < 0.05).
